# Circadian rhythm in hypothalamic leptin receptor (Ob-Rb) mRNA expressions and cerebrospinal fluid and circulating glucose and leptin levels in lactating rats

**DOI:** 10.1016/j.bbrep.2021.101129

**Published:** 2021-09-08

**Authors:** Yoshihiro Suzuki, Kiyohiko Takagishi, Yohei Kurose

**Affiliations:** Department of Animal Science, School of Veterinary Medicine, Kitasato University, Towada, Japan

**Keywords:** Leptin, Receptor, Cerebrospinal fluid, Glucose, Food intake, Lactation

## Abstract

In lactating animals, the food consumption increases several-fold for milk supply to the pups. The present study was conducted to clarify the relationship between the hyperphagia during lactation and hypothalamic leptin receptor (Ob-Rb) mRNA expression, cerebrospinal fluid (CSF) and circulating leptin and glucose levels. Food intakes significantly higher in lactation than in non-lactation at all time points (3 points: light phase, 4 points: dark phase) of the day. However, the expression of the hypothalamic Ob-Rb mRNA showed similar circadian rhythms in both the non-lactation and lactation, with only slight differences between the two groups. CSF leptin and glucose levels were constant throughout the day in both non-lactation and lactation, and there was almost no difference between the two groups at each time point. Circulating leptin and glucose levels showed circadian rhythms only in the non-lactating period, and were lower in lactation than in non-lactation, especially in the dark phase. In conclusion, the present study provides evidence that Ob-Rb mRNA expression fluctuates in the lactation period as well as in the non-lactation period, suggesting that the expression profile of whole hypothalamic Ob-Rb may not contribute to the difference in food consumption between lactation and non-lactation, and that chronic decrease in blood glucose levels may be associated with the increase in food consumption during lactation.

## Highlights

・ There was no difference in CSF leptin levels between lactating and non-lactating rats.
・ Hypothalamic Ob-Rb mRNA expressions showed different circadian rhythms between non-lactation and lactation.
・ Chronic decrease in blood glucose levels may be associated with the elevation of food consumption in lactating rats.

## 1 Introduction

In lactating animals, the food consumption increases several-fold for milk supply to the pups [1]. The rats as nocturnal animals eat mainly in the dark period. However, lactating rats eat much even in the light period as well as in the dark period [1], suggesting the loss of circadian rhythm of feeding behavior.

Glucose is the most important energy source for mammals. In addition, blood glucose is the most important resource for milk production in lactating animals. There is a close relationship between blood glucose levels and hypothalamic neural activity. During hypoglycemia, the blood glucose level is improved by the actions of the pancreas and the central nervous system [2]. Activation of the hypothalamic proopiomelanocortin and melanocortin 4 receptor neurons is essential for hypoglycemia counterregulatory [3]. In contrast, high glucose level in the brain results in hypothalamic leptin resistance in rats [4]. Lowered insulin sensitivity and leptin resistance induced by high-fat-high-sucrose dieting are accompanied by down-regulation of hypothalamic OB-Rb expression in rats [5].

Leptin is a hormone primarily secreted from the white adipose tissue (WAT). The effects of leptin have been mainly reported in the field of appetite, energy expenditure and reproduction such as sexual maturation and cycle [6]. In lactation, chronic peripheral administration of leptin didn't suppress food consumption [7]. On the other hand, chronic intracerebroventricular (i.c.v.) administration of leptin from day 8–15 of lactation reduced food intake [8]. Our previous study on lactating rats demonstrated that i.c.v. administration of leptin suppressed food intake, but that intravenous administration of leptin did not during 4 h after the onset of the dark period [9]. These results suggest that leptin resistance during lactation may develop only at the peripheral level. The hypothalamus is the highest center of the autonomic nervous system that can sense changes in both blood and CSF leptin levels [10,11]. Most of leptin's effects, including an anorectic effect, are mediated by its binding to the hypothalamic Ob-Rb [12]. Hypothalamic Ob-Rb mRNA expression shows a circadian rhythm in rats. In male ad libitum-fed rats, whole hypothalamic Ob-Rb gene expression, like that of adipocyte leptin gene expression, rose abruptly at the onset of nocturnal feeding behavior but receded progressively to low range thereafter [13]. On the other hand, the circadian rhythm of the arcuate nucleus (ARC) leptin sensitivity, which declining during the light phase, and increasing during the dark phase, was confirmed by a single intraperitoneal leptin injection to male mice after 24 h of fasting [14]. These reports show that rodents exhibit circadian rhythms within the hypothalamus for both leptin sensitivity and OB-Rb mRNA expression, although there are differences in feeding conditions (ad libitum-fed or 24 h of fasting) and measurement sites (whole hypothalamus or ARC). Therefore, the circadian rhythm of the hypothalamic OB-Rb mRNA expression may be involved in the feeding-inhibitory effect of leptin under ad libitum fed conditions in female rats.

Leptin has a greater anorectic effect on female rats than on male rats [15], suggesting that the expressions of Ob-Rb in female rats differ to those in male rats. Furthermore, we hypothesized that hypothalamic OB-Rb mRNA expression may contribute to the difference in food consumption between lactating and non-lactating female rats. However, there are few detailed analyses on the circadian rhythm of hypothalamic Ob-Rb mRNA expression in female rats as well as lactating rats. In our previous study, the plasma leptin levels of lactating rats were significantly lower than those of non-lactating female rats in the light period, but the expressions of hypothalamic Ob-Rb mRNA were similar [16]. Whereas, it has been reported that daytime hypothalamic Ob-Rb mRNA levels in lactation are significantly lower than those in non-lactation, and the normal nocturnal decreases in the expression of Ob-Rb were lost [17]. To clarify the reasons for these different results, the details of circadian rhythms during lactation and non-lactation need to be elucidated.

The purpose of present study is to elucidate the participation of hypothalamic Ob-Rb expression in the feeding regulations of lactating rats. To attain this purpose, we also focused on central glucose and leptin levels as a factor influencing the anorectic effect of leptin. In the present study, therefore, we examined the circadian rhythm of hypothalamic Ob-Rb mRNA expression and CSF glucose and leptin levels in non-lactating and lactating rats.

## 2 Materials and methods

### 2.1 Animals

All experimental procedures involving animals were performed in according to the guidelines on handling and care of animals by the committee for animal welfare of Kitasato University. Female Wistar rats of 10–17 weeks of age (CLEA Japan Inc., Japan) were used. The animals were housed under controlled lighting (12 h light: 12 h darkness cycle, light was turned on at 0600 h) and temperature (23 ± 1 °C) conditions with free access to food and water. The control animals (non-lactating) were virgin, in the diestrus stage of the estrous cycle, and of the same age as the lactating rats. Female rats were mated at 10 weeks of age. The litter size was adjusted to 10 pups (5 males and 5 females) at birth. Pups were weaned on day 21 of lactation.

### 2.2 Experiment 1: Changes in dams daily body weight, food intake, and plasma glucose and leptin level from pregnancy to lactation

Wistar female rats of 10 weeks of age were used for mating (n = 5). The data were serially collected from the same animals. Daily food intakes and body weights of dams were basically measured at 4-day intervals until day 21 of lactation (day of weaning). Blood samples were collected at 0900 h from dams in heparinized capillary tubes by the tail tip incision method. Blood samples were centrifuged at 4 °C, and plasma samples were stored at −30 °C until analysis of plasma leptin levels.

### 2.3 Experiment 2: The circadian rhythm of the hypothalamic Ob-Rb mRNA expression, CSF and plasma leptin levels, CSF and blood glucose levels, and food intakes on non-lactating and day 10 of lactating female rats

In experiment 1, increased daily food consumption in lactating rats, reached a plateau at day 9 of lactation. In experiment 2, therefore, we analyzed diurnal changes in food intake, CSF and blood glucose levels, CSF and plasma leptin levels, and hypothalamic Ob-Rb mRNA expression at day 10 of lactation to clarify factors related to the hyperphagia in lactation.

Data were obtained by euthanizing Wistar female rats of 14–15 weeks of age at 0600, 1000, 1400, 1800, 2100, 2400 and 0300 h for both non-lactation and day 10 of lactation (each point: n = 4–7). The intervals between food intake measurements were 4 h in the light period and 3 h in the dark period, and food intakes on non-lactation and day 10 of lactation were measured only for the time period preceding the individual being euthanized. After the measurement of food intake for 3 or 4 h, lactating and non-lactating rat were anaesthetized with pentobarbital sodium (40 mg/kg body weight), and blood (4 ml/each) and CSF (20 μl/each) samples were immediately obtained by puncturing the inferior vena cava and the cisterna magna, respectively. After blood collection, the blood glucose levels were measured immediately. The plasma was obtained by centrifugation. Brain samples were collected at each time point and hypothalamic block was immediately cut out from the brain. The hypothalamic blocks were frozen in liquid nitrogen. Plasma, CSF, and the hypothalamic blocks were stored at −80 °C until analyzed.

### 2.4 Analysis of CSF, plasma, and blood glucose concentration

In experiment 1, plasma glucose was determined using a FUJIFILM DRI-CHEM NX500V (Fuji Film Co., Japan). In the experiment 2, on the other hand, CSF and blood glucose were immediately measured using a glucose analyzer (GLU-1, TOA Electronics, Japan) which more sensitive instrument.

### 2.5 Analysis of CSF and plasma leptin concentration

In experiment 1, plasma leptin were determined using a rat leptin ELISA kit (Morinaga Institute of Biological Science, Inc., Japan) following each of the manufacturer's protocols. In experiment 2, on the other hand, CSF and plasma leptin were determined using rat leptin ELISA kit (YK051, Yanaihara Institute Inc., Japan) which more high sensitivity ELISA kit.

### 2.6 Real-time RT-PCR analysis

Total RNA was extracted from hypothalamic block by FastPure RNA kit (TAKARA BIO INC., Japan) according to the manufacturer's instructions. The ratio and concentration of the RNA was confirmed using absorbance. Total RNA samples were stored at −80 °C until analyzed. Hypothalamic block total RNA (100 ng in a final volume of 20 μl) were denatured at 37 °C for 15 min and 85 °C for 5 s then reverse transcribed to complementary DNA (cDNA) using PrimeScript RT regent kit (TAKARA BIO INC., Japan) on a thermal cycler (Mini Opticon, Bio-Rad Laboratories Inc., Japan). The primer sequence and the predicted size of the PCR products are shown in Table 1. Primer pairs for rat Ob-Rb were designed using Primer3 software [18] and BLAST [19], and those for the rat beta actin (Actb) primer as a housekeeping reference gene was purchased (TAKARA BIO INC., Japan). The cDNA primers to specific rat Ob-Rb and Actb were positioned to span intron-exon junctions to distinguish cDNA from genomic DNA.

**Table 1:** PCR primers and PCR products for Ob-Rb and Actb.

Primer		Sequence (5′-3′)	Product size (bp)	Accession number
**Ob-Rb**				
	Sense (1254–1275)	ccgctacgctgaattatatgtg	193	AF287268.1
	Antisense (1423–1446)	ctctgatgtaggacgaatagatgg		

**Actb**				
	Sence (1026–1048)	ggagattactgccctggctccta	150	NM031144.2
	Antisense (1152–1175)	gactcatcgtactcctgcttgctg		

Real-time PCR was carried out using SYBR Green I sequence non-specific detection. For amplification of cDNA, PCR was performed in total volume of 20 μl, made from 1.6 μl template cDNA, 10 μl SYBR Premix Ex Taq™ (TAKARA BIO INC., Japan), 0.8 μl of 10 μM sense and anti-sense primer mix and 7.6 μl dH_2_O on a thermal cycler (Mini Opticon, Bio-Rad Laboratories Inc., Japan). The amplification program was as follows: denaturation step (95 °C for 10 s) and 45 cycles of three step amplification (denaturation, 95 °C for 10 s; annealing, 58 °C for 5 s; and extension, 72 °C for 10 s). In order to verify the purity of the products, a melting curve was produced after each run by increasing the temperature of the reaction mixtures up to 95 °C, by 0.2 °C/2 s, starting 55 °C. Values for the threshold (Ct) were determined using the LightCycler software. Relative gene expression numbers were calculated with the Actb as reference gene. The hypothalamic Ob-Rb mRNA level at 0600 h in non-lactating female rats was assigned a value of 1.

### 2.7 Statistical analysis

All data were expressed as a mean with S.E.M. In experiment 1, chronological changes in body weight, food intake, and plasma glucose and leptin levels until weaning were analyzed by Dunnett's test. In experiment 2, the significance of difference in food intake between non-lactation and lactation during each period was analyzed by Student's t-test. Changes in leptin and glucose levels in blood, plasma, and CSF, and the hypothalamic Ob-Rb mRNA expressions were analyzed using 2-way ANOVA (lactation status and time), followed by Tukey's multiple comparison test.

## 3 Results

### 3.1 Experiment 1

**Fig. 1:**
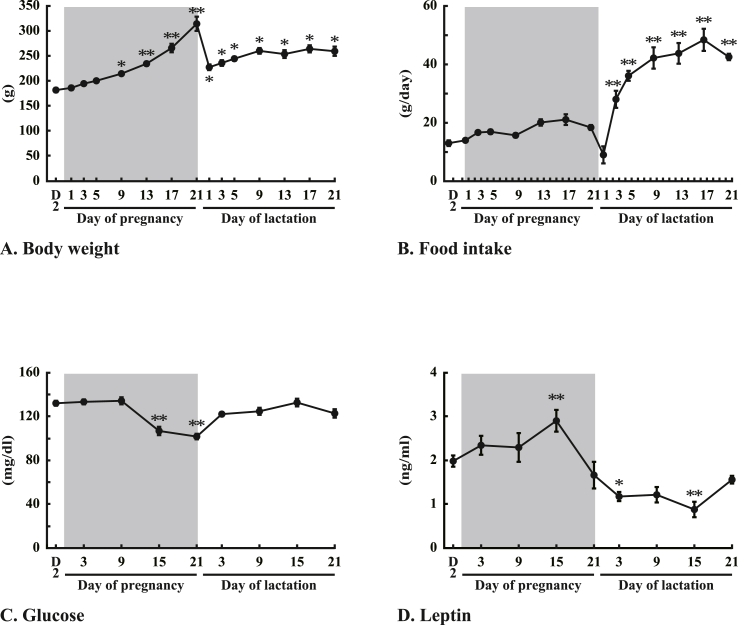
Changes in dams (A): body weight, (B): food intake for 24 h, (C): plasma glucose levels, and (D): plasma leptin levels from pregnancy to lactation. The shaded band represents the pregnancy period. Values are the means and vertical lines represent the S.E.M. (n = 5). *P < 0.05 and **P < 0.01 vs. diestrus 2 (D2) rats.

Body weights of dams from day 9 of pregnancy onward were significantly (P < 0.01 and P < 0.05) increased than those on diestrus 2 just before pregnancy (Fig. 1A). Food intakes during days 2–21 of lactation were significantly (P < 0.01) greater than those on diestrus-2 (Fig. 1B). Plasma glucose levels on days 15 and 21 of pregnancy were significantly (P < 0.01) less than those on diestrus-2 (Fig. 1C). Plasma leptin levels on day 15 of pregnancy were significantly (P < 0.01) greater than those on diestrus 2, but those on day 3 and 15 of lactation were significantly (P < 0.01 and P < 0.05) less than those on diestrus-2 (Fig. 1D).

### 3.2 Experiment 2

**Fig. 2:**
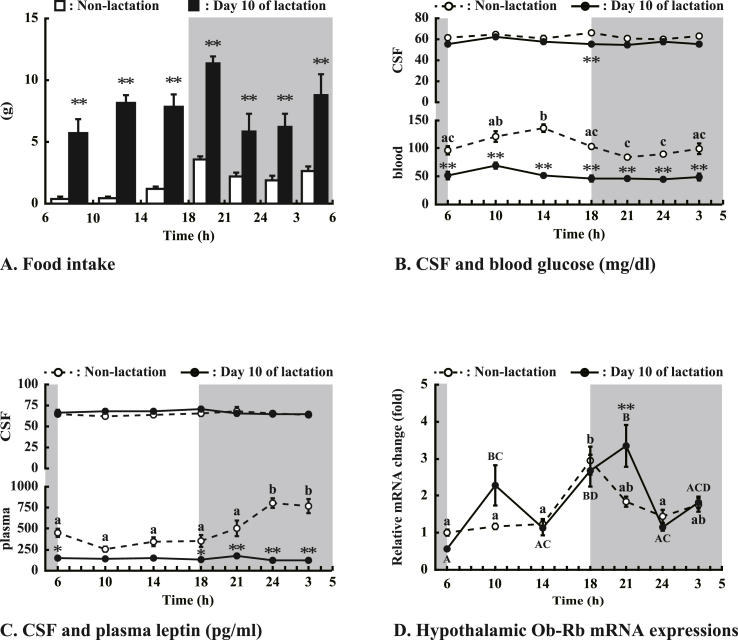
Circadian rhythm of (A): food intake, (B): CSF and blood glucose levels, (C): CSF and plasma leptin levels, and (D): hypothalamic OB-Rb mRNA expressions in non-lactation and day 10 of lactation. Shaded band represents the dark (light-off) phase. Values are the means and vertical lines represent the S.E.M. (n = 4–7/time point). Dissimilar superscripts denote significant differences (non-lactation; a,b, and c P < 0.05, lactation; A,B,C, and D P < 0.05). *P < 0.05 and **P < 0.01 vs. non-lactation at the same time.

The results of 2-way ANOVA showed that there was a significant interaction between physiological condition (lactation and non-lactation) and time in blood glucose levels, plasma leptin levels, and hypothalamic Ob-Rb mRNA expression (P < 0.01). Fig. 2A shows diurnal variations of food consumption in non-lactation and lactation. Across all time periods, food intakes of the lactating rats were significantly (P < 0.01) greater than those of the non-lactating rats. Fig. 2B shows changes of CSF and blood glucose levels in non-lactation and lactation. CSF glucose kept a constant level throughout the day in non-lactation and lactation. CSF glucose levels in lactation were significantly (P < 0.01) less than those in non-lactation at 1800 h. Blood glucose levels significantly (P < 0.05) increased in the light phase (1000 and 1400 h) in non-lactation but not in lactation. Blood glucose levels in lactation were significantly (P < 0.01) less than those in non-lactation at all time points. Fig. 2C shows changes of CSF and plasma leptin levels in non-lactation and lactation. CSF leptin kept a constant level across the light and dark phase in both physiological conditions. Furthermore, there was no difference in CSF leptin levels between non-lactation and lactation. On the other hand, plasma leptin levels significantly (P < 0.05) increased at 2400 and 0300 h in non-lactation but kept constant in lactation. Plasma leptin levels in lactation were significantly (P < 0.01 and P < 0.05) less than those in non-lactation at 0600, 1800, 2100, 2400, and 0300 h. Hypothalamic Ob-Rb mRNA expressions showed different diurnal variations between non-lactation and lactation (Fig. 2D). In non-lactating rats, the hypothalamic Ob-Rb mRNA expression just after the onset of the dark phase (1800 h) was significantly (P < 0.05) higher than those at other times. In lactating rats, hypothalamic Ob-Rb mRNA expression reached the maximum level at 2100 h, significantly (P < 0.05) higher than that at 0600, 1400, 2400 and 0300 h. In addition, the hypothalamic Ob-Rb mRNA expression at 1800 h was significantly (P < 0.05) higher than that at 0600, 1400, and 2400 h in lactating rats. The hypothalamic Ob-Rb mRNA expressions in lactation were significantly greater (P < 0.01) than those in non-lactation at 2100 h.

## 4 Discussion

In experiment 1, plasma glucose levels decreased during late pregnancy. The blood glucose levels during mid- and late-pregnancy are significantly lower than those in non-lactating female rats [20]. To efficiently supply nutrients to the fetus, various maternal physiological changes, such as blood sex hormone levels, development of placenta and insulin resistance, occur as the pregnancy progresses [21]. In this study, however, there was no difference in the daily food consumption between the pregnant and the non-pregnant (diestrus-2) rats. Therefore, the decrease in plasma glucose levels from mid-to late-pregnancy may be due to increased nutrient requirement of the developing fetus. There was a difference in circulating glucose levels between experiment 1 and 2. It may be due to differences in blood collection sites (the inferior vena cava or tail vein), anesthesia, and pretreatment of blood samples (plasma or blood). In Experiment 2, blood was collected from inferior vena cava, so it is considered that the decrease in blood glucose levels reflects milk production in the mammary gland. Across all time periods, food intakes of the lactating rats were greater than those of the non-lactating rats. The most possible explanation for this is great demand of glucose to maintain active milk synthesis. In fact, blood glucose levels of the lactating rats were kept lower than those of the non-lactating rats regardless of high food consumption. On the other hand, despite hyperphagia at all time points during lactation, there was no difference in CSF glucose levels at most time points between the non-lactation and lactation. It has been reported that rapid inhibition of glucose uptake by single intraperitoneal injection of 2-deoxy-d-glucose on day 10 of lactating rats did not increase short-term food intake [22]. These results suggest that hyperphagia during lactation may not be induced by short-term decreases in CSF glucose level, but may be related to chronic decreases in peripheral glucose levels.

As has been reported by our previous study [9], the plasma leptin levels of the lactating rats were less than those of the non-lactating female rats, whereas the CSF leptin levels had no difference between both rats while maintaining a steady level. Furthermore, the nocturnal rise in plasma leptin was attenuated in the lactating rats as has been reported [17]. These results suggest that the increase in circulating leptin levels, that occurs after feeding, is suppressed during lactation. In our previous study, we have confirmed that the plasma leptin levels in dams on day 1 after weaning were suddenly increased relative to the day before weaning [16]. Woodside et al. [8] reported that when milk secretion during lactation was stopped by galactophore-cuts prior to mating, plasma leptin levels in operated dams increased several times more than those in sham operated dams. Therefore, milk synthesis and suckling stimulation may be involved in the decrease in circulating leptin levels during lactation. From the late pregnancy to lactation, blood prolactin levels of dams are increased compared with those in non-lactating female [23]. In addition, prolactin inhibits insulin-dependent leptin production in WAT [24]. In the present study, however, leptin did not decrease in the late pregnancy when circulating prolactin and placental lactogen levels are generally elevated. It has been reported that insulin sensitivity is lower in the late pregnancy than in lactation [25]. Therefore, circulating leptin levels in the late pregnancy may be kept by non-insulin-dependent mechanisms and thus free from the inhibitory effects of prolactin.

We have clearly demonstrated diurnal fluctuations of hypothalamic Ob-Rb mRNA expression exist in both non-lactating and lactating female rats as has been reported in male rats [13]. Hypothalamic Ob-Rb mRNA expression was elevated at the onset of the dark phase when feeding behavior becomes active in both non-lactating and lactating female rats. However, the elevation of hypothalamic Ob-Rb mRNA expression was prolonged into the dark phase in the lactating rats. The elevation of hypothalamic Ob-Rb mRNA expression during the nocturnal feeding period is inconsistent with the anorectic property of leptin. In mice, hypothalamic Ob-Rb mRNA expression is increased by fasting, and is returned by simultaneous intraperitoneal injection of insulin and leptin [26]. In the lactation period, therefore, the prolonged elevation of Ob-Rb mRNA in the dark phase may be partly due to low levels of plasma insulin, leptin, and blood glucose.

The suprachiasmatic nuclei (SCN) in the hypothalamus is the central control site that regulates circadian rhythms in the body through light information, and is controlled by a group of major clock genes: *Clock*, *Per*, *Bmal1*, and *Cry*. When the SCN of rats is disrupted, the circadian rhythm of feeding behavior disappears, and many feeding behaviors have been observed even in the light period [27]. Feeding behavior during lactation was not associated with the light-dark cycle. In mouse, in addition, the expression of SCN *Per2* mRNA in lactation was higher than that in pregnancy at middle of light and dark periods [28]. Therefore, the circadian rhythms of major clock genes in SCN may be strongly associated with hyperphagia and hypothalamic OB-Rb mRNA expression during lactation. Further studies are needed to elucidate these relationships. In addition, as has been mentioned above, the elevation of hypothalamic Ob-Rb mRNA expression is inconsistent with the elevation of food consumption especially in the lactating rats, suggesting that leptin signaling pathway for suppressing food consumption may be inhibited in the lactating rats. Prolactin-responsive cells that express the leptin receptors distribute in the hypothalamus [29]. During late pregnancy and lactation, prolactin increases the ARC suppressor of cytokine signaling 3 (SOCS3) mRNA expression [30]. This SOCS3 is one of the signal inhibitors of leptin receptors [31]. In lactation period, therefore, suppression of leptin signaling pathway may be caused by PRL-R expression in the hypothalamus. To elucidate the discrepancy between feeding and the circadian rhythm of the whole hypothalamic Ob-Rb mRNA expressions in lactating rats, further studies are necessary to examine more detailed hypothalamic regions such as ARC, paraventricular nucleus (PVN), supraoptic nucleus (SON), and SCN.

In conclusion, the results of this study provide evidence that decreased blood glucose levels may be associated with the increase in food consumption during lactation. Furthermore, it has been clearly shown that Ob-Rb mRNA expression fluctuates in the lactation period as well as the non-lactation period. However, it is suggested that the expression profile of whole hypothalamic Ob-Rb may not contribute to the difference in food consumption between lactation and non-lactation. Further studies are necessary to examine more detailed hypothalamic regions such as ARC, PVN, SON, and SCN instead of the whole hypothalamus.

## Declarations of competing interest

The authors declare that there is no conflict of interest that could be perceived as prejudicing the impartiality of the research reported.
